# Abscisic Acid and Sulfate Offer a Possible Explanation for Differences in Physiological Drought Response of Two Maize Near-Isolines

**DOI:** 10.3390/plants9121713

**Published:** 2020-12-05

**Authors:** Avat Shekoofa, Thomas R. Sinclair

**Affiliations:** 1 West TN Research & Education Center, Plant Sciences Department, University of Tennessee, Jackson, TN 38301, USA; 2 Crop and Soil Sciences Department, North Carolina State University, Raleigh, NC 27695-7620, USA; trsincla@ncsu.edu

**Keywords:** maize near-isolines, abscisic acid, sulfate, osmotic potential

## Abstract

The hypothesis was tested that differences in response to water-deficits between low osmotic potential (LOP) and high osmotic potential (HOP) maize (*Zea mays* L.) near-isolines were associated with differences in transpiration rate sensitivity to abscisic acid (ABA) and/or sulfate. In a series of four experiments, decreases in transpiration rate (DTR) of whole plants and fully expanded leaves were measured in response to treatments of 1.0 µM ABA and 15 mM MgSO_4_ singly and in combination following long (2 day) and short (180 min) exposures. There was little evidence that intact plants grown on soil were responsive to the treatments. For hydroponically grown plants subjected to long exposure, there was similarly no response to treatments. Further, the short exposure of hydroponically grown plants to solely ABA or a combination of chemicals resulted in no sensitivity in DTR for either of the near-isolines. On the other hand, when these plants were fed sulfate, the transpiration was stimulated by about 20% for the LOP and 60% for the HOP. Detached leaves proved to be the most sensitive to treatment. Treatment with the two chemicals singly caused essentially equivalent DTR in the two near-isolines. However, treatment with ABA plus sulfate resulted in different DTR between the two near-isolines with values of 65% for the LOP and 16% for the HOP near-isoline. Overall, these results showed that the short exposure treatment of hydroponically grown plants or detached leaves supported the hypothesis of different transpiration rate sensitivities of the near-isolines in response to ABA and sulfate treatments.

## 1. Introduction

One of the most common abiotic stresses suffered by plants is drought. Soil water deficits develop in the absence of water input as a consequence of transpirational water loss, which is quantitatively linked to stomatal opening to allow CO_2_ to diffuse into leaves [1]. The stomatal aperture is in turn necessarily matched closely to the hydraulic flow of water from roots into leaves [2] to avoid leaf desiccation.

A major component in setting the leaf transpiration rate is the gradient of water vapor pressure between the interior of the leaves and the atmosphere, which is identified as the vapor pressure deficit (VPD). The water vapor pressure of the air space within the leaf is close to saturation. So a dry atmosphere, i.e., low vapor pressure, gives rise to a large gradient in water vapor, i.e., VPD, across the stomatal pore. Limiting transpiration by partial stomatal closure, especially early in the crop growing season and high VPD conditions, will allow plants to conserve soil water for use when drought may develop. This limited-transpiration (TR_lim_) trait in response to high VPD has been observed in the genotypes of several crop species [3,4,5,6,7,8].

In addition to partial stomatal closure in response to VPD, partial closure occurs in response to drying soil. This response is now frequently expressed as the decreased plant normalized transpiration rate (NTR) at a low fraction of transpirable soil water (FTSW) during a soil drying cycle. Possible differences among genotypes in sensitivity to soil drying can be quantified by the FTSW at which the breakpoint for the decline in NTR is initiated. Genotypes with higher FTSW breakpoints are more conservative in their soil water use, and consequently, these genotypes are likely better suited for arid environments [8,9].

As an alternative to the two water-conservation approaches described above, greater water extraction has been proposed for plants having lower osmotic potential (OP) [10,11,12], resulting in a greater hydraulic gradient for more rapid or additional water uptake from the soil [13]. This approach was studied in two inbred near-isolines of maize (*Zea mays* L.), which had different levels of expression of leaf osmotic potential under water-deficit conditions in the field [14]. It was found that the near-isoline with the lower OP had greater leaf area duration, water extraction at soil depths greater than 0.80 m, and grain yield. In controlled-environment studies, Beseli et al. [8] found that the maize near-isoline with the lower (more negative) osmotic potential (LOP) in the early stages of the soil drying cycle had the greatest transpiration rate and delayed wilting, which was consistent with the original field observations. Visual wilting was observed in the LOP plants 4 days after wilting developed in the high osmotic potential (HOP) near-isoline. However, the endpoint of water extraction supporting transpiration (NTR ≤ 0.1) occurred 5 days earlier in the LOP near-isoline than the HOP near-isoline.

Interestingly, the two near-isolines also differed in their expression of the two water-conservation traits [8]. In response to VPD, the LOP near-isoline was found to have a breakpoint in the transpiration response at 2.7 kPa, while the HOP showed no breakpoint. In response to soil drying, the threshold for decrease in NTR was consistently at a lower FTSW for the LOP line than the HOP line.

In contrast to the hydraulic perspective in the studies with the two maize near-isolines, much drought-related research has focused on water-deficit stress as it relates to the hormone abscisic acid (ABA) and its connection to stomatal closing. A number of studies have reported that drought stimulates the production of chemical signals in the roots of plants, which are then hypothetically transported to the shoots to regulate stomatal behavior [15,16]. Abscisic acid is a candidate for this chemical signal since it has been shown in independent studies to be synthesized in roots under drought stress [17,18], transported to the shoot in xylem sap [19,20], and entered into guard cells to induce stomatal closure [21]. Numerous experimental approaches have implicated the involvement of ABA in root-to-shoot signaling [22], but the nature of the signaling pathway for ABA from roots to stomata is still poorly understood.

A previous study with maize indicated that not only root-sourced ABA but also xylem-born ABA and sulfate were involved in maize transpiration rate decreases under water-deficit stress [21]. Ernst et al. [21] found that the anti-transpirant effect of ABA at the early stage of water deficit was enhanced by the increased levels of sulfate also transported in the xylem. Batool et al. [23] tested the impact of increasing sulfate concentrations on the maize guard cell in detached leaves that were fed with sulfate. Increasing sulfate concentrations from 0 to 2 mM caused stomatal closure (*p* < 0.05).

To make progress in resolving the ABA/sulfate hypothesis for regulating the stomata aperture, it could be especially beneficial to study the response of the maize near-isolines documented to respond differently to water deficit. Specifically, a series of experiments was undertaken to explore this hypothesis that the differences in response to high VPD and soil-water deficit between the two maize near-isolines originate from differences in sensitivity to ABA and/or sulfate. The first set of experiments tested the possible differential sensitivity of the two near-isolines to ABA and sulfate by applying ABA and/or sulfate for several days to the soil on which intact plants were growing. A companion experiment was done by transferring hydroponically grown plants to solutions containing the chemical treatments to avoid any confounding influence of applying treatments to soil. Transpiration rate was measured over a range of VPD since the near-isolines were reported to have differential sensitivity to VPD. The second set of experiments were done via short-term exposures of hydroponically grown plants to ABA and/or sulfate treatments. The third set of experiments tested detached leaves to examine specifically the sensitivity of the leaves of the near-isolines to the feeding of the chemicals.

## 2. Results

A preliminary test was done with the detached leaves of both near-isolines to confirm they were both sensitive to the 1.0 µM (for ABA) and 15 mM (for MgSO_4_) concentrations reported by Ernest et al. [21]. In fact, sensitivity of the transpiration rate to these concentrations was found for both near-isolines, and it was subsequently confirmed in a detached leaf experiment that both near-isolines were sensitive singly to 1.0 µM ABA and 15 mM MgSO_4_, and to the combination of the two (data not shown, but confirmed in current study’s figures).

### 2.1. Long Exposure to Chemicals

#### 2.1.1. Intact Plants Grown on Soil

The impact of chemical treatment on the response of transpiration rate to VPD was tested on soil-grown plants. For the HOP near-isoline, a linear increase in transpiration with increasing VPD was observed for all treatments (Table 1), as originally reported for soil-grown HOP plants by Beseli et al. [8]. There was no difference among treatments in the slope of this linear response.

The LOP plants grown on soil, which were reported by Beseli et al. [8] to have a breakpoint response with increasing VPD, did not express a breakpoint following the two treatments with sulfate. While a breakpoint response was observed following the ABA treatment, the breakpoint was at a low value of 1.24 kPa as compared to the value of 2.20 kPa reported by Beseli et al. [8]. The slope of the transpiration rate vs. VPD under low VPD was much greater for the ABA treatment than was observed for the single linear slope found in all other cases (Table 1), although the standard error of this slope was very high. The very low breakpoint and high variability in the slope following the ABA treatment raises the possibility that the breakpoint response might be an analytical artefact. That is, all chemical treatments applied to the soil may have resulted in a loss of the breakpoint response of the LOP near-isoline.

#### 2.1.2. Intact Plants Grown Hydroponically

The influence on transpiration rate of chemical treatment was determined on plants grown hydroponically to eliminate any confounding effect of applying the treatment to soil. The HOP near-isoline again showed a linear response of TR to varying VPD following all chemical treatments. There was no difference among chemical treatments in the slope of this linear response (Table 1).

The LOP subjected to the water-only treatment expressed the two-segment response (Table 1) originally reported for this near-isoline by Beseli et al. [8]. In fact, the parameters of the response function were very similar to those of Beseli et al. The two treatments with sulfate also showed the breakpoint response, although the slopes at VPD above the breakpoint VPD were substantially negative. These results indicate an amplified closure of the stomata due to sulfate under high VPD (Table 1).

In contrast to the other treatments of the LOP near-isoline, the ABA treatment resulted in the expression of a linear response over the entire range of tested VPD (Table 1). This result indicates that ABA may have entered the plant from the hydroponic solution and interfered with the expression of a breakpoint response at elevated VPD.

### 2.2. Short Exposure to Chemicals

#### 2.2.1. Intact Plants

**Figure 1:**
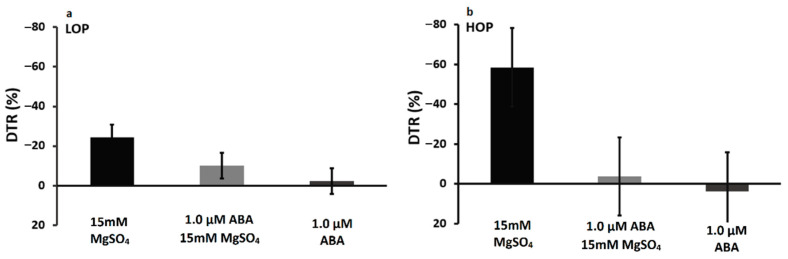
Decrease in the transpiration response (DTR) of intact plants of two maize near-isolines LOP (**a**) and HOP (**b**) at 32/26 °C and high VPD ~3.2 kPa. The treatments were (ABA (1.0 µM), MgSO_4_ (15 mM), and a combination (ABA (1.0 µM) + MgSO_4_ (15 mM))). The intact plants were grown hydroponically.

In contrast to the delay of one or two days in the measurement of transpiration response to the chemical treatments in the previous experiments, this experiment was designed to measure response after a short exposure to the treatments. For both near-isolines, there was little change in transpiration rate, i.e., DTR near zero, when ABA was applied either by itself or in combination with sulfate (Figure 1). Unexpectedly, both LOP and HOP near-isolines had stimulated water loss rates (negative DTR) in response to exposure to sulfate. The increase in intact-plant water loss was greater in the HOP line as compared with the LOP line (*p* value = 0.03).

#### 2.2.2. Detached Leaves

**Figure 2:**
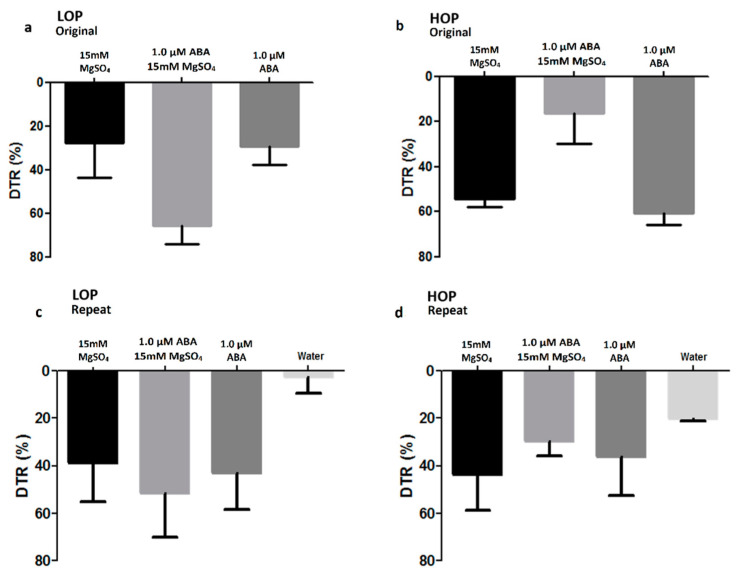
Decrease in transpiration response (DTR) of the detached leaves of two maize near-isolines LOP (**a**,**c**) and HOP (**b**,**d**) at 32/26 °C and high VPD ~3.2 kPa. The treatments were (ABA (1.0 µM), MgSO_4_ (15 mM), and a combination (ABA (1.0 µM) + MgSO_4_ (15 mM))). The plants grown on soil.

Measurements of the transpiration response of detached leaves to chemical treatments were done to give the most direct measure of leaf transpiration response to the chemical treatment. A significant decrease was observed in transpiration rate when 1.0 µM ABA and 15 mM MgSO_4_ were applied individually to both near-isolines (Figure 2). That is, within each near-isoline, both chemical treatments resulted in substantial decreases in transpiration rate, that is, a large DTR value.

The two maize near-isolines differed, however, in their response to the combination treatment of ABA plus sulfate as compared to the individual treatments (Figure 2). For the LOP line in the original experiment, the combination of both chemicals resulted in a greater DTR than either of the chemicals given singularly (*p* value = 0.006 and 0.008 for sulfate and ABA vs. the combined treatment, respectively) (Figure 2a). In the repeat experiment, the DTR for the LOP line was significantly greater when detached leaves were fed ABA (*p* value = 0.006), MgSO_4_ (*p* value = 0.01) and the combination (*p* value = 0.0007) compared to deionized water (Figure 2c). However, in the repeat experiment there were no differences in the LOP near-isoline for the DTR of ABA vs. the combination (*p* value = 0.85) and sulfate vs. the combination (*p* value = 0.63) (Figure 2c).

In contrast to the LOP near-isoline, the combination treatment of ABA plus sulfate in the HOP near-isoline resulted in a significantly lower DTR in the original experiment than was observed in either of the chemicals applied individually (*p* value = 0.0008 and 0.0005 sulfate and ABA vs. the combined treatment, respectively) (Figure 2b). In the repeat experiment of the HOP near-isoline (Figure 2d), however, high *p* values were obtained in DTR for ABA vs. deionized water (*p* value = 0.38), MgSO_4_ vs. deionized water (*p* value = 0.12), and the combined treatment vs. deionized water (*p* value = 0.79). High *p* values were also obtained in the repeat experiment in the comparison of ABA vs. a combination of chemicals (*p* value = 0.95), and sulfate vs. a combination of chemicals (*p* value = 0.94).

When the combination treatments of ABA plus sulfate of the two experiments (i.e., Figure 2 original and repeat) were averaged and compared between the two near-isolines (i.e., LOP vs. HOP), the DTR of the LOP near-isoline was significantly greater than the HOP near-isoline (*p* value = 0.0003).

## 3. Discussion

Chimenti et al. [14] showed that the maize near-isolines they developed expressed differing responses to water-deficit treatments in the field, with the LOP near-isoline being more resilient than the HOP near-isoline until the stress became severe. Beseli et al. [8] showed the near-isolines also differed in their response to elevated VPD and soil water content. The LOP near-isoline had a two-segment response to VPD and partial stomatal closure at lower soil water content than the HOP near-isoline. In the current study, through a series of experiments the hypothesis was examined that the basis for the expression of the physiological differences between the near-isolines results from a difference between the near-isolines in transpiration rate sensitivity to ABA and/or sulfate.

When intact plants were grown on soil and the soil was treated with 1.0 µM ABA, 15 mM sulfate, or both in combination, there was little evidence that the plants were responsive to the treatments (Table 1). A linear increase in transpiration rate with increasing VPD was found for all the treatments of the HOP near-isoline. A two-segment response was generated by the statistical analysis for the ABA treatment of the LOP near-isoline, but the nature of the result indicated that the result could have been an artefact.

Since the application of the chemical treatments to soil could have confounded the results, a subsequent experiment was done with hydroponically grown plants. The comparison of near-isoline plants tested on hydroponically grown plants matched the overall observations of Beseli et al. [8]. That is, in all treatments, the HOP near-isoline showed the linear response of the transpiration rate to VPD, and the LOP near-isoline showed the two-segment response except for in the treatment with only ABA (Table 1). The exceptional case indicates that the ABA treatment overcame the limitation in the LOP plant that caused partial stomatal closure at a VPD of 2.2 kPa in plants supplied only with water. Since the prevalent hypothesis is that ABA induces stomatal closure [20,21], the results obtained for the LOP near-isoline indicated there may be an alternative site of ABA sensitivity. One possibility is that the ABA in this experiment may have increased the root hydraulic conductance in the LOP near-isoline so that there was no limitation of water flow, and consequently, there was no stomatal closure at elevated VPD.

In the measurement of short exposure to the treatment of hydroponically-grown intact plants, treatments of solely 1.0 µM ABA and a combination of chemicals resulted in essentially no change in transpiration rate, i.e., a DTR near zero for both near-isolines (Figure 1). However, the sulfate treatment resulted in increases in transpiration rate for both near-isolines, with the HOP having much higher increases (more negative DTR) than the LOP (*p* value = 0.03). The stimulation of transpiration rate in the sulfate treatment in the short-exposure experiment differed from the response of the hydroponically grown plants in the long-exposure experiment, in which the HOP near-isoline was insensitive to sulfate and the LOP near-isoline expressed a limited transpiration rate at an elevated VPD. These contrasting results indicate that there was adjustment in plants exposed for a longer duration to sulfate. Such a hypothesized adjustment indicates that the results from short-term experiments may be inappropriate in reflecting the activity of sulfate and/or ABA in plants subjected to the development of water deficits over long time-periods in the field.

The detached leaf experiments showed the substantial sensitivity of the transpiration rate to treatment with ABA and sulfate (Figure 2), as has been previously reported in individual genotypes [21,24]. In each of the tests, the impact of ABA and sulfate given individually was equal. However, a differential response in DTR was observed with the combination treatment of ABA plus sulfate as compared to the individual chemical treatments. Particularly interesting was the fact that the DTR response to the combined chemicals was opposite between the two near-isolines. The combined treatments caused the transpiration rate to decrease to a much greater extent in the LOP near-isoline than in the HOP near-isoline. This greater sensitivity of the DTR of the LOP near-isoline to the combination of ABA and sulfate would be consistent with sensitivity to stress as a result of elevated VPD. That is, the results of the detached-leaf experiments support the hypothesis that the LOP near-isoline is more sensitive to a combination of ABA and sulfate than the HOP near-isoline, and this may cause the phenotypic difference in plant response to VPD.

## 4. Materials and Methods

### 4.1. Plant Material

The two near-isolines (S4 populations) developed by Chimenti et al. [14] from Argentinean flint germplasm and adapted to temperate environments were used in this study. Parents of these lines were chosen from a group of 20 elite inbred maize lines, provided by the Maize Breeding Program of Pergamino Experiment Station-INTA (National Institute of Agricultural Technology, Buenos Aires, Argentina). Two sets of maize families were developed by selection of differential response to an osmotic test. This resulted in two breeding populations of near-isogenic background with differing levels of osmotic adjustment [14].

The sensitivity of the transpiration rate of the near-isolines to the ABA and SO_4_ treatments was tested on four-week-old plants. Plants were grown on soil in the first experiment and hydroponically for two additional experiments (Table 2). A fourth experiment was done using leaves detached from plants.

In all experiments, three chemical treatments were imposed: 1.0 µM ABA, 15 mM MgSO_4_, and a combination of ABA and MgSO_4_ (1.0 µM + 15 mM). Ernest et al. [21] observed a decrease in transpiration as the treatment concentration of ABA increased from 0.3 to 1.0 µM. Additionally, a higher concentration of ABA such as 1.0 µM resulted in further decreases in transpiration rate when combined with sulfate [21,23]. The combination of 1.0 µM ABA and 15 mM for MgSO_4_ was especially potent in decreasing stomatal aperture of Vicia faba [21].

### 4.2. Long Exposure to Chemicals

#### 4.2.1. Intact Plants Grown on Soil

Maize seedlings were grown in pots constructed from polyvinyl chloride pipe 10 cm in diameter and 25 cm tall. The plants were grown on topsoil substrate (#92432, Lowes Inc., N. Wilkesboro, NC, USA), which included 14:6.1:10 (N:P_2_O_5_:K_2_O) fertilizer. The plants were grown in the greenhouse for 3 to 4 weeks with daily watering to maintain well-watered conditions. However, the plants were not watered on the day that chemical solutions were applied to the soil to avoid the confounding influence of hypoxia. When the maize plants developed four fully expanded leaves, the plant response to the application of the chemical treatments was measured. On the afternoon prior to making the measurements, instead of watering the pots, 250 mL (i.e., pot capacity) of chemical solution treatment was applied to the soil surface of each pot.

The transpiration response to chemical treatment over a range of VPDs was measured in an enclosed system similar to that described by Fletcher et al. [3]. The pots in which the plants had been grown had a toilet flange attached at their tops. When transpiration measurements were to be made, a 340 mm diameter food container lid (Cambro Manufacturing, Huntington Beach, CA, USA) with the center cut out was attached to the toilet flange. The soil surface around the plant was sealed with aluminum foil to prevent soil evaporation. The following morning the aerial part of each plant was enclosed in a 21 L clear plastic food container (Cambro Manufacturing, Huntington Beach, CA, USA) by placing the inverted container over the plant and attaching it to the previously installed lid. Each container, that is, VPD chamber, was fitted with a 12 V, 76 mm diameter computer box fan (Northern Tool and Equipment, Burnsville, MN, USA) to continuously stir the air inside the chamber. In addition, a humidity/temperature data logger (Lascar Electronics, Erie, PA, USA) was mounted through the sidewall of each container to measure the chamber environment.

Transpiration rates were measured gravimetrically on two consecutive days under different levels of VPD, from low to high (0.5–3.5 kPa). On each day, the maize plants were exposed to three levels of atmospheric VPD: low (0.5–1.5 kPa), medium (2.0–2.5 kPa) and high (3.0–3.5 kPa). Plants were allowed 30 min for acclimation at each VPD and then the pot weight loss over 1 h was measured to obtain transpiration rate. After completing measurements on the second day, the plants were harvested. Leaves were separated from the stem, and leaf area was measured using a leaf area meter (LI-1300, Licor, Lincoln, NE, USA). Transpiration rate was expressed on a plant leaf area basis.

The observations of each near-isoline in each treatment were combined in graphs of transpiration rate vs. VPD. The results were analyzed using a two-segment linear regression analysis (Prism 7.0, GraphPad, Software Inc., San Diego, CA, USA) of transpiration rate vs. chamber VPD. If the slopes of the two segments were not different at *p* ≤ 0.05, a simple linear regression was applied to all the data.

#### 4.2.2. Intact Plants Grown Hydroponically

This hydroponic experiment was done using pots (polyvinyl chloride pipe 10 cm in diameter and 25 cm tall) that had been fitted with a flat end. A toilet flange was also affixed to the top of these pots to allow the easy attachment of the VPD chamber during measurements. A 7.5 cm thick piece of foam (Multi-Purpose Foam, The Home Depot, Raleigh, NC, USA) was installed in the mouth of each pot. A small cut in the center of each piece of foam allowed a plant to be held and the insertion of an air tube for solution aeration. An air pump was used to flow air into the hydroponic solution of each pot at 1 L min^−1^. The hydroponic solution during the growth of the plants was the standard solution used in the North Carolina State University Phytotron [25]: MgSO_4_ (0.3 mM), K_2_NO_3_ (2.0 mM), CaSO_4_ (0.8 mM), KH_2_PO_4_ (0.6 mM), Fe-Sequestrene (36 μM), H_3_BO_3_ (0.61 μM), MnCl_2_ (0.12 μM), ZnSO_4_ (0.11 μM), CuSO_4_ (0.13 μM), and Na_2_MoO_4_ (0.003 μM). The hydroponic growth solution was changed every other day. Solution pH was measured between 5.5–6.0. The plants were grown in a walk-in growth chamber with day and night temperatures of 32 and 26 °C, respectively.

Once the hydroponically grown plants had developed four fully expanded leaves, the plants were transferred the day before measuring transpiration response to identical pots containing the chemical treatments dissolved in deionized water. The switch from nutrient solution to chemicals dissolved in deionized water was to eliminate any possible interactions between the target chemicals and cations/ions in the nutrient solution. As done in the experiment with plants grown on soil, transpiration rates were measured on the next two days after treatment over a wide range of humidity (i.e., VPD 0.5–3.5 kPa).

### 4.3. Short Exposure to Chemicals

#### 4.3.1. Intact Plants

This short-exposure experiment was designed to measure a nearly immediate response to the chemical treatments of intact plants. For this experiment, the plants were again grown hydroponically. Five day-old maize seedlings were each mounted in rubber stoppers and placed on 1 L Erlenmeyer glass flasks containing the same hydroponic solution as described previously. The rubber stopper had two holes; one hole to hold the plant and the other to allow access of an air tube for solution aeration. An air pump was used to flow air continuously into the nutrient solution of each flask at 0.5 L min^−1^. The plants were grown in a greenhouse with day and night temperatures of 32 and 26 °C, respectively. The hydroponic growth solution was changed every other day. On the alternate days, deionized water was added to each flask daily to replace water losses. The recorded solution pH was between 5.5 and 6.0.

Once the plants had developed four fully expanded leaves, they were transferred to a walk-in growth chamber on the day before transpiration measurements were to be made. The plants were transferred to aerated flasks containing only deionized water. The temperature in the growth chamber was held between 31 and 32 °C during the measurements. The air of the growth chamber was dried by using a dehumidifier to achieve a VPD of 3.1 kPa, which was greater than the VPD breakpoint reported by Beseli et al. [8] for the LOP near-isoline.

On the following day, the plants were allowed 60 min to acclimatize after the lights were turned on (500 to 550 μmol m^−2^ s^−1^ at plant level) before measurements were initiated. Following acclimatization, the flask plus plant was weighed. After 120 min, the flask plus plant was reweighed and the difference between the two weights divided by the time interval was used to calculate initial transpiration rate in deionized water (TR_0_). After the second weighing, all plants were quickly transferred and sealed into new 1 L flasks containing one of the three chemical treatments (i.e., ABA (1.0 µM) and MgSO_4_ (15 mM) singly, and in combination (1.0 µM ABA +15 mM MgSO_4_)). After allowing 60 min to reach a new steady-state water loss rate, the flasks plus plants were weighed to obtain initial treatment weights. After an interval of 180 min, the flasks plus plants were reweighed to allow the calculation of transpiration rate for the chemical treatment [26,27]. The transpiration rate following exposure to ABA, sulfate_,_ and their combination (TR_x_) was calculated based on the difference between these two weights.

The decrease in transpiration rate (DTR) for each individual plant as a result of the chemical treatments was calculated using the following Equation (1):

DTR = 100 × (TR_o_ − TR_x_)/TR_o_ (1)

Therefore, small TR_x_ rates due to chemical treatment resulted in large values of DTR. In each experiment, Tukey’s HSD test was performed to compare the decrease in transpiration rate (DTR) response between the two isolines. Software packages JMP 13, Prism 7.0 and GraphPad were used for the statistical analysis and for creating graphs.

#### 4.3.2. Detached Leaves

Maize plants were grown in a greenhouse (32/26 °C day/night) in pots (2.3 L) filled with Gardenplus topsoil (#92432, Lowes Inc., N. Wilkesboro, NC, USA), which included 14:6.1:10 (N:P_2_O_5_:K_2_O) fertilizer. Five seeds were sown in each pot and later thinned to four plants in each pot. Pots were maintained in a well-watered condition by watering twice a day. At approximately 4 weeks, the leaves from the plants were harvested.

On the evening of the day prior to the experiment, fully developed maize leaves were gently cut from the leaf sheath below the collar. The detached leaves were immediately placed in a container filled with deionized water and a second cut was made for each leaf underwater above the collar to minimize embolism. Then, the leaves were placed in individual 120 mL flasks filled with deionized water (6 replications per each near-isoline). The flasks with the detached leaves were moved to a walk-in growth chamber and the flasks were sealed with Para-film to avoid direct water evaporation. The detached leaves were kept under 26 °C in the dark until the following morning [9,27].

After the lights were turned on the next day, the detached leaves were allowed to acclimatize for 30 min to the photosynthetic photon flux density of 500 to 550 μmol m^−2^ s^−1^ at plant height. The VPD in the growth chamber was maintained between 3.0 and 3.2 kPa (i.e., high VPD). Following acclimatization, the flasks plus the detached leaves were weighed for an initial weight. After 120 min, the flasks were reweighed, and the difference between the two weights divided by the time interval was used to calculate TR_0_. Following the second weighing of the detached leaves in water, the individual leaves were immediately transferred to 50 mL glass bottles for exposure to solutions of 1.0 µM ABA and 15 mM MgSO_4_ singly, and in combination (1.0 µM + 15 mM). The detached leaves were allowed to take up the solutions for 60 min, by which time the transpiration rate of the leaves had again reached a constant value [26,27], then all the bottles were weighed to get an initial weight for the transpiration measurement following exposure to the chemical treatments. After 180 min, each bottle was again weighed. The TR_x_ following exposure to ABA, MgSO_4_, and their combination was calculated based on the difference between these two weights. The decrease in transpiration rate (DTR) was calculated the same as described above for the intact plants. The original experiment was repeated with the addition of a deionized water-only treatment.

## 5. Conclusions

This series of experiments was done to examine the hypothesis that differences in transpiration response to high VPD and water-deficit between the two maize near-isolines are associated with differences in transpiration rate sensitivity to ABA and/or sulfate. No significant differences were found among the chemical treatments for the HOP near-isoline in both long-exposure experiments. In the LOP near-isoline grown hydroponically, the ABA treatment resulted in the expression of a linear transpiration response over the entire range of tested VPD. In short-term studies with intact plants, there was little change in transpiration rate, i.e., DTR near zero, when ABA was applied either singly or in combination with sulfate. In contrast, the transpiration rate was stimulated by the sulfate treatment in both near-isolines with the HOP near-isoline having greater stimulation than the LOP near-isoline. These results indicate an immediate differential transpiration rate sensitivity between the near-isolines to sulfate. In the detached leaf experiment, the combination treatment of ABA plus sulfate resulted in a much greater decrease in transpiration rate in the LOP near-isoline as compared to the HOP near-isoline. This difference in the transpiration rate’s sensitivity to the combination treatment was consistent with the expression of a limited transpiration at elevated VPD by the LOP near-isoline. The observations of chemical sensitivity in the short experiments were not apparent in the long experiments. This raises the possibility that short-term experiments with ABA may be inappropriate for reflecting the activity of ABA and/or sulfate in plants for longer-term experiments appropriate for field interpretation.

## Figures and Tables

**Table 1:** Whole plant transpiration response to ABA (1.0 µM), MgSO_4_ (15 mM), and a combination (1.0 µM + 15 mM) application under varying levels of VPD in two days (long-term chemical treatments). Results from two-segment linear regression include Slope 1 (±SE), 95% Confidence Interval of Slope 1, Slope 2 (±SE), BP, and their R^2^. The results of those fitted by a single linear regression include the slope and R^2^.

Experiment							
Maize Isolines	TR vs. VPD Soil Substrate			TR vs. VPD Hydroponic Substrate			
HOP	ABA (1.0 µM)	MgSO_4_ (15 mM)	ABA + MgSO_4_ (1.0 µM + 15 mM)	ABA (1.0 µM)	MgSO_4_ (15 mM)	ABA + MgSO_4_ (1.0 µM + 15 mM)	Water
**Slope 1 ± SE ^a^**	15.23 ± 2.34	15.12 ± 2.86	17.33 ± 1.40	21.56 ± 2.49	18.45 ± 2.36	16.06 ± 1.64	12.16 ± 1.04
**Slope 1-** **95% Confidence Interval**	10.0 to 20.4	8.7 to 21.5	14.2 to 20.4	16.3 to 26.7	13.5 to 23.3	12.6 to 19.4	10.0 to 14.3
**Slope 2 ± SE**	-	-	-	-	-	-	-
**BP ^b^**	-	-	-	-	-	-	-
**R^2^**	0.80	0.73	0.93	0.77	0.73	0.81	0.86
**LOP**							
**Slope 1 ± SE**	30.77 ± 12.2	14.95 ± 2.18	12.79 ± 2.01	14.24 ± 1.90	29.78 ± 6.42	23.19 ± 4.14	16.68 ± 3.71
**Slope 1-** **95% Confidence Interval**	2.59 to 59.0	8.29 to 17.2	10.0 to 19.8	10.2 to 18.1	16.3 to 43.1	14.5 to 31.8	8.87 to 24.4
**Slope 2 ± SE**	9.33 ± 2.23	-	-	-	−15.34 ± 13.38	−38.09 ± 50.86	7.85 ± 3.72
**BP**	1.24 ± 0.35	-	-	-	2.41 ± 0.264	2.60 ± 0.44	2.20 ± 0.62
**R^2^**	0.97	0.82	0.80	0.71	0.62	0.66	0.83

^a^ The standard error (SE) is given for each of the outputs from the regression analyses. ^b^ BP: VPD breakpoint.

**Table 2:** Description of the conditions for the list of experiments plus the duration of each experiment.

Experiment Description	TR vs. VPD		DTR (%)	
	Long Exposure/Intact Plant		Short Exposure	
	Soil	Hydroponic	Intact Plant	Detached Leaf
**Pot or flask volume**	1.4 L Pot	1.4 L Pot	1.0 L Erlenmeyer flask	2.3 L Pot
**Substrate during growth**	Garden plus topsoil	Hydroponic solution [25]	Hydroponic solution [25]	Garden plus topsoil
**Substrate during experiment**	1.0 µM ABA 15 mM MgSO_4_ (1.0 µM + 15 mM)	1.0 µM ABA 15 mM MgSO_4_ (1.0 µM + 15 mM)	1.0 µM ABA 15 mM MgSO_4_ (1.0 µM + 15 mM)	Deionized water 1.0 µM ABA 15 mM MgSO_4_ (1.0 µM + 15 mM)
**Environment during growth**	Walk-in growth chamber	Walk-in growth chamber	Greenhouse	Greenhouse
**Environment during experiment**	21 L chamber inside a walk-in growth chamber	21 L chamber inside a walk-in growth chamber	Walk-in growth chamber	Walk-in growth chamber
**VPD (kPa)**	0.5–3.5	0.5–3.5	3.0–3.2	3.0–3.2
**Duration of growth**	30 d	30 d	29 d	29 d
**Duration of chemical treatments**	2 d *	2 d	180 min **	180 min
**Duration of experiment**	2 d	2 d	1 d	1 d

* Long term chemical treatments (i.e., 2 days). ** Short term chemical treatments (i.e., 180 min).

## Abbreviations

ABA	Abscisic acid
VPD	Vapor pressure deficit
OP	Osmotic potential
LOP	Low osmotic potential
HOP	High osmotic potential
TR	Transpiration rate
DTR	Decreases in transpiration rate
FTSW	Fraction of transpirable soil water
NTR	Normalized transpiration rate
